# Smad3 Mediates Diabetic Dyslipidemia and Fatty Liver in db/db Mice by Targeting PPARδ

**DOI:** 10.3390/ijms241411396

**Published:** 2023-07-13

**Authors:** Huijun He, Yu Zhong, Honglian Wang, Patrick Ming-Kuen Tang, Vivian Weiwen Xue, Xiaocui Chen, Jiaoyi Chen, Xiaoru Huang, Cheng Wang, Huiyao Lan

**Affiliations:** 1 Division of Nephrology, Department of Medicine, The Fifth Affiliated Hospital of Sun Yat-sen University, Zhuhai 519000, China; hehuijun@link.cuhk.edu.hk; 2 Department of Medicine and Therapeutics, Li Ka Shing Institute of Health Sciences, The Chinese University of Hong Kong, Hong Kong 999077, China; yuzhong@link.cuhk.edu.hk (Y.Z.); honglianwang@swmu.edu.cn (H.W.); chenxiaocui2012@outlook.com (X.C.); margaret.chenjy@link.cuhk.edu.hk (J.C.); xlan@cuhk.edu.hk (X.H.); 3 Guangdong Provincial Key Laboratory of Biomedical Imaging, The Fifth Affiliated Hospital of Sun Yat-sen University, Zhuhai 519000, China; 4 State Key Laboratory of Translational Oncology, Department of Anatomical and Cellular Pathology, The Chinese University of Hong Kong, Hong Kong 999077, China; patrick.tang@cuhk.edu.hk (P.M.-K.T.); vivian.xue@connect.polyu.hk (V.W.X.)

**Keywords:** Smad3, diabetes, dyslipidemia, fatty liver, Smad3 inhibitor, treatment

## Abstract

Transforming growth factor-β (TGF-β)/Smad3 signaling has been shown to play important roles in fibrotic and inflammatory diseases. However, the role of Smad3 in dyslipidemia and non-alcoholic fatty liver disease (NAFLD) in type 2 diabetes remains unclear, and whether targeting Smad3 has a therapeutic effect on these metabolic abnormalities remains unexplored. These topics were investigated in this study in Smad3 knockout (KO)-*db/db* mice and by treating *db/db* mice with a Smad3-specific inhibitor SIS3. Compared to Smad3 wild-type (WT)-*db/db* mice, Smad3 KO-*db/db* mice were protected against dyslipidemia and NAFLD. Similarly, treatment of *db/db* mice with SIS3 at week 4 before the onset of type 2 diabetes until week 12 was capable of lowering blood glucose levels and improving diabetic dyslipidemia and NAFLD. In addition, using RNA-sequencing, the potential Smad3-target genes related to lipid metabolism was identified in the liver tissues of Smad3 KO/WT mice, and the regulatory mechanisms were investigated. Mechanistically, we uncovered that Smad3 targeted peroxisome proliferator-activated receptor delta (PPARδ) to induce dyslipidemia and NAFLD in *db/db* mice, which was improved by genetically deleting and pharmacologically inhibiting Smad3.

## 1. Introduction

Dyslipidemia is highly prevalent among type 2 diabetes (T2D). It is characterized by elevated triglycerides (TG), decreased levels of high-density lipoprotein cholesterol (HDL-C), and elevated low-density lipoprotein cholesterol (LDL-C) and small, dense LDL particles [1,2,3]. Many studies have demonstrated that hyperglycemia and dyslipidemia are risk factors of cardiovascular diseases (CVD) and significantly contribute to the development of diabetic complications, including cardiovascular and kidney diseases in patients with T2D [3,4,5,6,7]. In addition, increased TG/HDL-C ratio and serum levels of TG can also predict the development of insulin resistance [8]. It has been reported that insulin resistance with an attendant increase in free fatty acid flux into the liver may play a central role in promoting diabetic dyslipidemia [1,2,3]. Moreover, increased free fatty acid flux can promote hepatic triglyceride production, which also contributes to the development of non-alcoholic fatty liver disease (NAFLD) [9,10,11]. However, the underlying mechanism of diabetic dyslipidemia is complex and only partially understood. Thus, research into the mechanisms of dyslipidemia and NAFLD under diabetic conditions is the first step toward the development of effective treatment for diabetes-associated dyslipidemia and NAFLD.

Transforming growth factor-β (TGF-β)/Smad3 signaling is highly activated and plays a regulatory role in diabetes and diabetic complications, which has been demonstrated by deleting Smad3 from *db/db* mice or by overexpressing renal Smad7 in *db/db* mice to protect against the development of T2D and diabetic complications including type-2 diabetic nephropathy (T2DN) and myocardial disease [12,13,14,15,16,17,18]. Thus, targeting TGF-β/Smad signaling by overexpressing Smad7 or inhibiting Smad3 has been shown to have therapeutic effects on T2D and T2DN [17,18]. In addition, treatment with a Smad3-specific inhibitor SIS3 can also significantly improves streptozotocin (STZ)-induced type 1 diabetic nephropathy [19]. However, the mechanisms through which Smad3 regulates diabetic dyslipidemia and NAFLD and, importantly, whether the direct inhibition of Smad3 has a therapeutic effect on diabetic dyslipidemia and NAFLD in T2D, remain largely unknown. These topics were investigated in the present study in Smad3 KO/WT-*db/db* mice and in *db/db* mice treated by SIS3.

## 2. Results

### 2.1. Smad3 Deficiency Inhibits Dyslipidemia and Fatty Liver in db/db Mice

**Figure 1:**
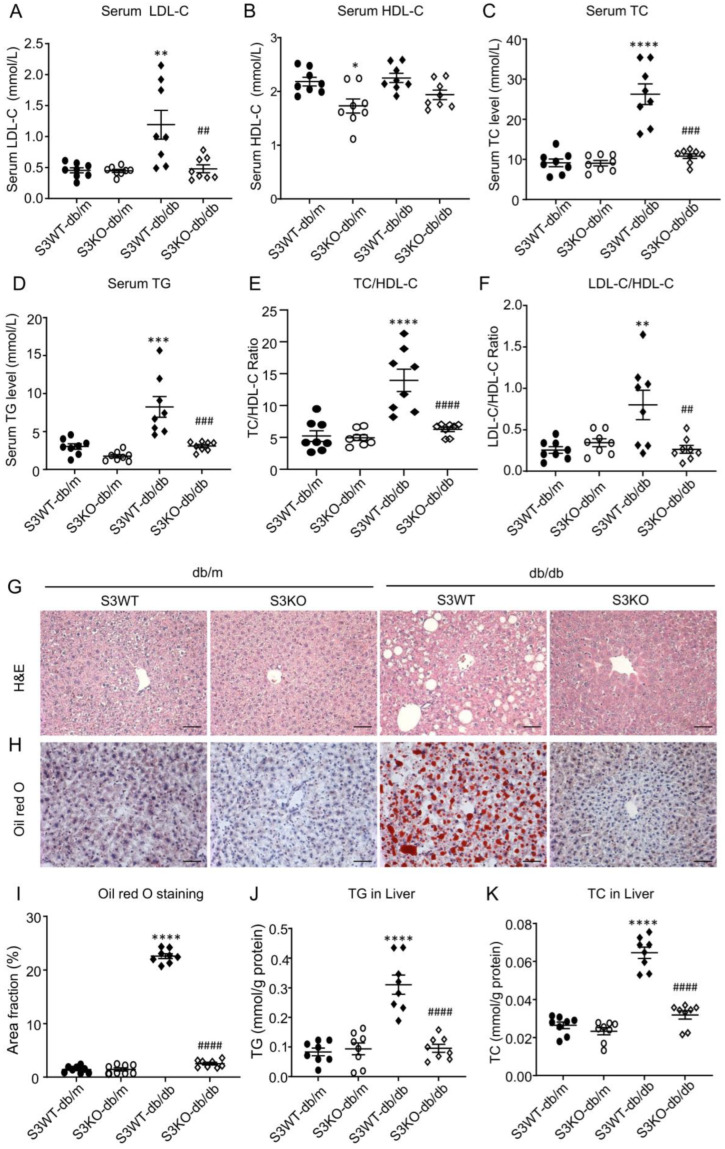
Smad3 deficiency attenuated hyperlipidemia and fatty liver in *db/db* mice. Smad3 deficiency reduced the serum levels of the LDL-C (**A**), TC (**C**), TG (**D**), TC/HDL-C ratio (**E**), LDL-C/HDL-C ratio (**F**), and improved fatty liver, as determined by H&E staining (**G**), Oil red O staining (**H**,**I**), TG accumulation (**J**), and TC accumulation (**K**). (**B**) Serum HDL-C levels of 4 groups of mice. Data represent the mean ± SEM for each group of at least 8 animals. The representative images are from at least three independent experiments. * *p* < 0.05, ** *p* < 0.01, *** *p* < 0.001, **** *p* < 0.001 versus normal db/m mice. ^##^ *p* < 0.01, ^###^ *p* < 0.001, ^####^ *p* < 0.0001 as indicated. Scale bar, 50 μm.

To explore the role of Smad3 in the onset of T2D-related dyslipidemia, we genetically deleted Smad3 from *db/db* mice (Smad3 KO-*db/db* mice) as previously described [13,14,15]. Compared to Smad3 WT-*db/db* mice, Smad3 KO-*db/db* mice were protected from the development of T2D, including hyperglycemia, glucose intolerance, insulin resistance, and T2DN and myocardial disease [13,14,15]. In the present study, we found that compared to Smad3 WT-*db/db* mice, Smad3 KO-*db/db* mice were also protected from the development of diabetic dyslipidemia as demonstrated by the normal serum levels of LDL-C level, total cholesterol (TC), TG, and the normal ratios of LDL-C/HDL-C and TC/HDL-C (Figure 1A–F). Interestingly, Smad3 deficiency also significantly reduced the serum HDL-C level in db/m mice but was only a trend reduction in Smad3 KO-*db/db* mice when compared with Smad3 WT-*db/db* mice (Figure 1B). We next examined whether the deletion of Smad3 prevents the development of fatty liver in *db/db* mice. As shown by H&E and Oil Red O staining, severe lipid accumulation in the liver tissue of Smad3 WT-*db/db* mice was almost completely inhibited in Smad3 KO-*db/db* mice (Figure 1G–I). Quantitative analysis further confirmed that the deletion of Smad3 from *db/db* mice completely inhibited a marked increase in the TG and TC contents in the liver tissues (Figure 1J,K). These results reveal that Smad3 is pathogenic in the development of dyslipidemia and NAFLD in T2D.

### 2.2. Deletion of Smad3 from db/db Mice Prevents the Development of Fatty Liver by Restoring PPARδ

**Figure 2:**
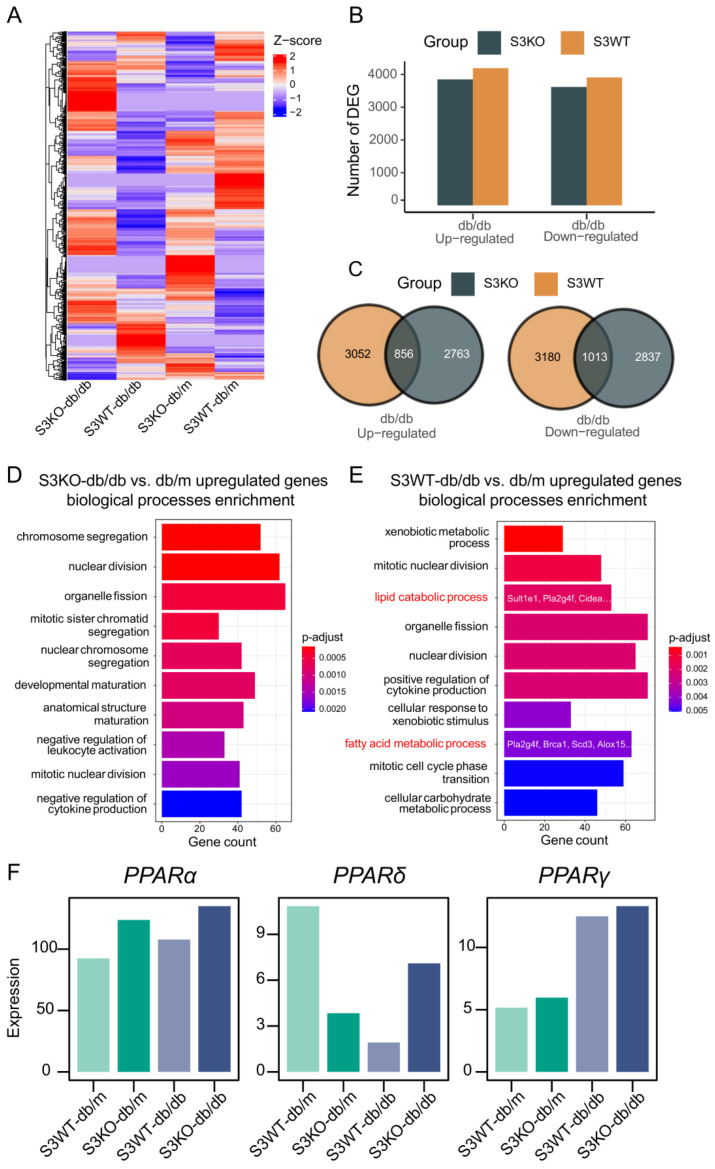
Smad3 deficiency attenuates fatty acid signaling genes in diabetic livers. (**A**) Heatmap showing the relative gene expression of each sample profiled by high throughput RNA sequencing. Each row in the heatmap correspond to expression of a gene. (**B**,**C**) Bar plot and Venn plot showing the differential expression genes number of *db/db* mice in Smad3 KO or Smad3 WT group. (**D**,**E**) The top 10 gene ontology (GO) biological process (BP) terms enrichment of increased genes in Smad3 WT- and Smad3 KO-*db/db* livers showing that increased genes in Smad3 WT-*db/db* were significantly enriched in lipid catabolic metabolism and fatty acid metabolism related pathways. (**F**) The relative expression of PPARs gene in four groups of mice.

**Figure 3:**
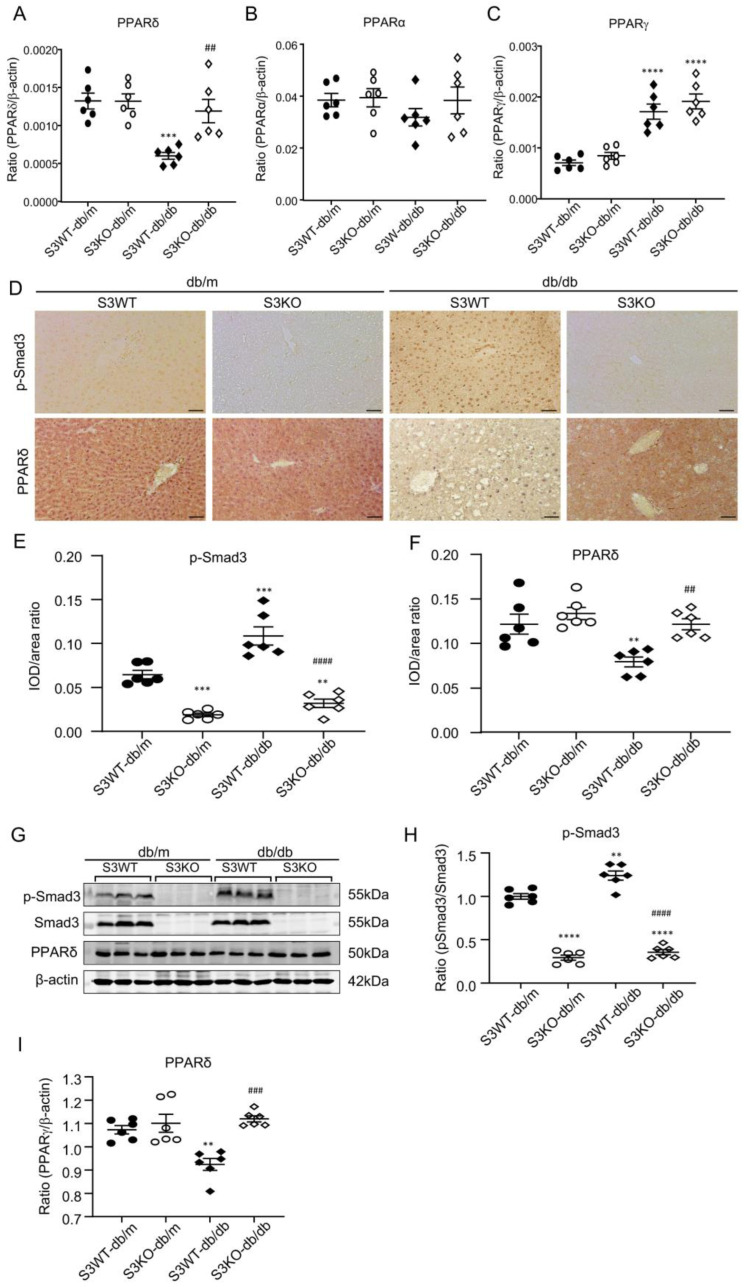
Smad3 deficiency restores PPARδ expression in liver tissues of *db/db* mice. (**A**–**C**) Liver PPARs levels detected by real-time PCR in Smad3 WT/KO-*db/m* or *db/db* mice. (**D**–**F**) Immunohistochemical staining and quantitative analysis of liver p-Smad3 and PPARδ in Smad3 WT/KO-db/m or *db/db* mice. (**G**–**I**) Western blotting analysis of liver p-Smad3, Smad3, PPARδ, β-actin in Smad3 KO/WT-db/m or *db/db* mice. Data represents the mean ± SEM for groups of 6 animals. ** *p* < 0.01, *** *p* < 0.001, **** *p* < 0.0001 versus normal db/m mice. ^##^ *p* < 0.01, ^###^ *p* < 0.001, ^####^ *p* < 0.0001, compared with the Smad3 WT-*db/db* mice. Scale bar, 50 μm.

We next examined the regulatory role of Smad3 in lipid metabolism by conducting lipid-related RNA-sequencing (RNA-seq) in the liver tissues obtained from Smad3 WT/KO-db/m and Smad3 WT/KO-*db/db* mice. There were 5600 Smad3-dependent genes that were differentially expressed in the liver tissues of *db/db* mice, whereas half of genes were reduced in Smad3 KO-*db/db* mice (Figure 2A–C). Gene ontology (GO) biological process (BP) terms enrichment revealed that compared to Smad3 KO-*db/db* mice, the increased genes in Smad3 WT-*db/db* livers were significantly enriched in the “lipid catabolic process” (*Sult1e1*, *Pla2g4f*, *Cidea*, *Pik3cg*, *Plcd3*, *Prdx6b*, *Naaa*, *Smpd3* et al.) and “fatty acid metabolic process” (*Pla2g4f*, *Brca1*, *Scd3*, *Alox15*, *Cyp2s1*, *Alox5ap*, *Cyp4a14*, *Acss1*, et al.), implying a promoting role of Smad3 in multiple lipid metabolism genes at the transcriptional level. Analysis of the up- and downregulated DEGs in the liver tissues of Smad3 KO-*db/db* relative to Smad3 WT-*db/db* mice also showed that genes reduced in Smad3 KO-*db/db* mice were significantly enriched in unsaturated fatty acid metabolic process (Figure S2A,B). This was associated with the enrichment of the inflammatory-related transcription factors (TFs) including *NFKB1*, *RELA*, *SPI1*, *JUN*, *ETS1* and *E2F1* (Figure S3). Since peroxisome proliferator-activated receptor (PPAR)s have been shown to play an important role in regulating lipid metabolism, NAFLD, and diabetic complications [20,21], we then analyzed the relative expression of PPARs genes in the liver tissues and found that among PPARs genes (*PPARα, PPARδ, and PPARγ*), only *PPARδ* was largely decreased in the liver tissues of Smad3 WT-*db/db* mice, which was reversed in Smad3 KO-*db/db* mice (Figure 2F), suggesting that *PPARδ* may be a key regulator of Smad3 in the development of diabetic dyslipidemia and NAFLD. This was further examined by real-time PCR. The data from RNA-seq showed that the deletion of Smad3 restored the normal level of liver *PPARδ* in Smad3 KO-*db/db* mice without altering the expression levels of *PPARα* and *PPARγ* (Figure 3A–C). Immunohistochemistry (IHC) staining and western blotting analysis also confirmed the notion that the deletion of Smad3 from *db/db* mice blocked Smad3 signaling and restored the expression of PPARδ to the normal level (Figure 3D–I).

### 2.3. Treatment with SIS3 Significantly Improves Diabetic Dyslipidemia in db/db Mice

**Figure 4:**
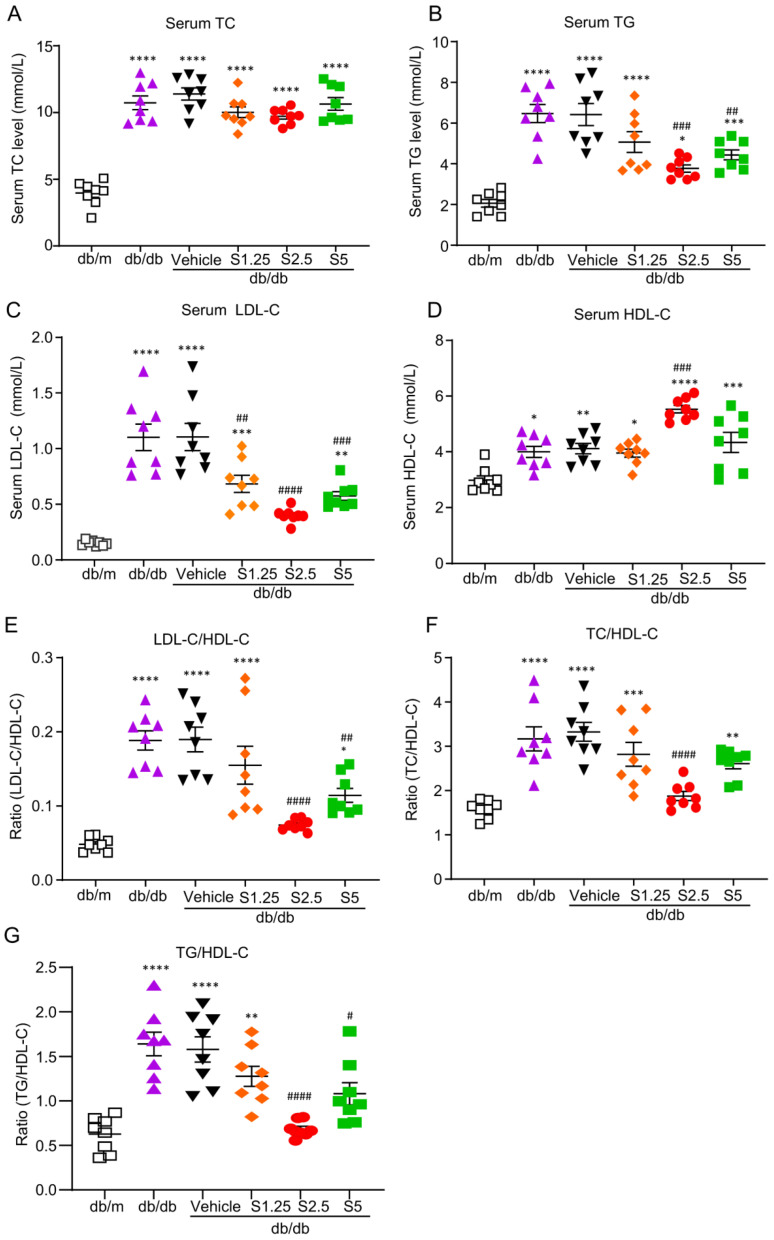
SIS3 treatment effectively ameliorated dyslipidemia in *db/db* mice. SIS3 treatment significantly reduced the (**B**) serum TG level, (**C**) serum LDL-C level, (**E**) serum LDL-C/HDL-C ratio, (**F**) TC/HDL-C ratio, and (**G**) TG/HDL-C ratio while increasing the serum HDL level (**D**) in *db/db* mice. However, SIS3 treatment did not change the serum TC level in *db/db* mice (**A**). Data represent the mean ± SEM for at least 8 mice per group. * *p* < 0.05, ** *p* < 0.01, *** *p* < 0.001, **** *p* < 0.001 versus normal db/m mice. ^#^ *p* < 0.05, ^##^ *p* < 0.01, ^###^ *p* < 0.001, ^####^ *p* < 0.0001 compared with the control-treated *db/db* mice (vehicle). SIS3 treatment dose: S1.25 = 1.25 mg/kg.bw, S2.5 = 2.5 mg/kg.bw, S5 = 5 mg/kg.bw.

Based on the results obtained from Smad3 KO-*db/db* mice that genetic deletion of Smad3 protected *db/db* from the development of dyslipidemia (Figure 1) and T2D [13], we then tested our hypothesis that targeting Smad3 may have a therapeutic effect on diabetic dyslipidemia and T2D by treating pre-diabetic *db/db* mice with a Smad3 inhibitor SIS3. We first determined an optimal therapeutic dose of SIS3 on diabetic dyslipidemia. Male *db/db* mice were treated with a daily SIS3 at dosages of 1.25, 2.5 and 5 mg/kg body weight intraperitoneally from the age of 4 weeks to 12 weeks. The results showed that treatment with SIS3 dose-dependently reduced serum levels of TG, LDL-C, and the ratios of LDL-C/HDL-C, TC/HDL-C, and TG/HDL-C, while increasing serum level of HDL-C in *db/db* mice (Figure 4). Of them, SIS3 at 2.5 mg/kg produced the best therapeutic effect on dyslipidemia (Figure 4). We then determined the therapeutic effects of SIS3 with the best dose at 2.5 mg/kg on T2D and found that compared with the control solvent-treated *db/db* mice (vehicle), treatment with SIS3 significantly reduced the levels of FBG and improved the intraperitoneal glucose tolerance test (IPGTT) and intraperitoneal insulin tolerance test (IPITT) (Table 1).

We also determined the systemic toxicity of SIS3 in db/m and *db/db* mice and found that treatment with SIS3 at 2.5 mg/kg showed no systemic toxicity, as demonstrated by normal levels of serum LDH and unchanged body weight (Table 2), without detectable abnormalities in the liver tissues of db/m mice (Figure S1A–C).

### 2.4. Treatment with SIS3 Significantly Attenuates Fatty Liver in T2D Mice

**Figure 5:**
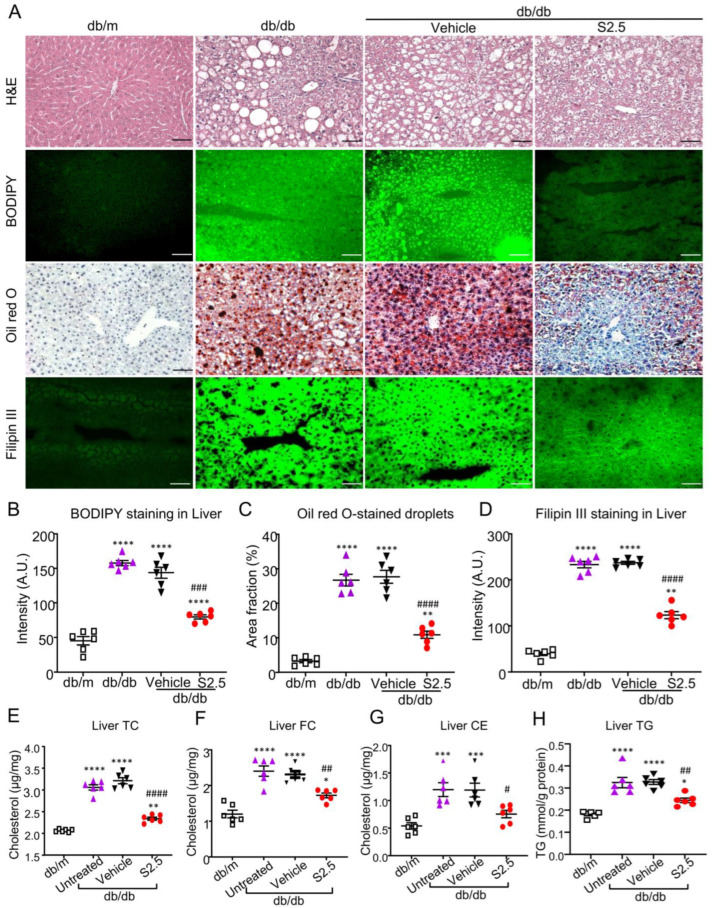
SIS3 treatment attenuated fatty liver in *db/db* mice. Compared with the control-treated *db/db* mice (vehicle), SIS3 treatment inhibited lipid disposition in the liver tissues (**A**), the lipid droplets’ area fraction and intensity (**B**–**D**), the liver total cholesterol level (**E**), the liver-free cholesterol level (**F**), the liver cholesteryl ester level (**G**), and the triglyceride level (**H**) in *db/db* mice. Data represent the mean ± SEM for at least 6 mice per group. * *p* < 0.05, ** *p* < 0.01, *** *p* < 0.001, **** *p* < 0.0001 versus normal db/m mice. ^#^ *p* < 0.05, ^##^ *p* < 0.01, ^###^ *p* < 0.001, ^####^ *p* < 0.0001 compared with the vehicle. Scale bar, 50 μm. SIS3 treatment dose: S2.5 = 2.5 mg/kg.bw.

We then determined the therapeutic effect of SIS3 on the development of diabetic fatty liver and found that treatment with SIS3 inhibited diabetic liver injury as evidenced by lowering serum levels of ALT and AST, as well as the liver weight, with the most effective dose at 2.5 mg/kg (Table 3 and Figure S1B,C). Further studies examining the liver lipid droplets contents by H&E staining, BODIPY (493/503) staining, Oil Red O staining, and Filipin III staining detected that treatment with SIS3 at an optimal dose of 2.5 mg/kg significantly reduced the lipid accumulation in the liver tissues of *db/db* mice (Figure 5A–D). Because liver is the main organ involving the cholesterol metabolism and previous studies have suggested that disturbed hepatic cholesterol homeostasis and liver free cholesterol accumulation contributes to the development of atherosclerotic dyslipidemia and NAFLD [22], we thus determined the therapeutic effect of SIS3 on liver lipid metabolism and found that SIS3 treatment significantly reduced TC, FC, cholesteryl esters (CE), and TG in *db/db* mice (Figure 5E–H).

### 2.5. Treatment with SIS3 Increases PPARδ Expression in the Liver Tissues of db/db Mice

**Figure 6:**
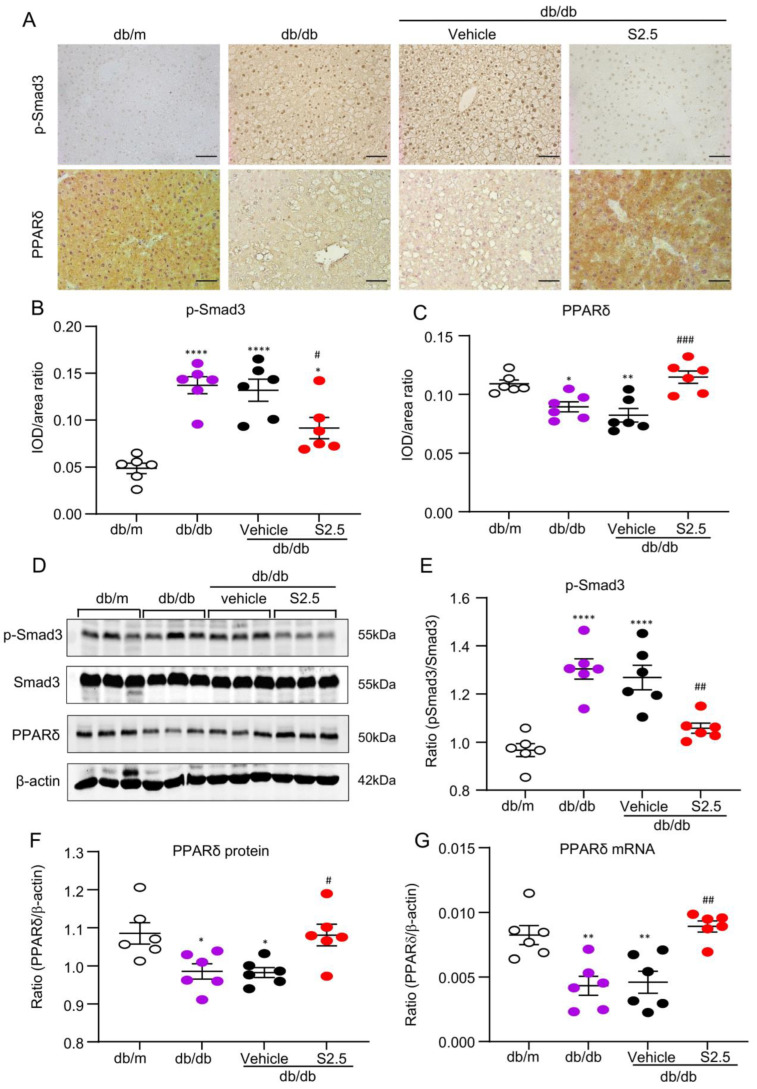
SIS3 treatment increased PPARδ expression in the liver tissues of *db/db* mice. (**A**–**C**) Immunohistochemical staining and quantitative analysis of p-Smad3 and PPARδ in the liver tissues. (**D**–**F**) Western blot analysis of p-Smad3 and PPARδ expression in the liver tissues. (**G**) Liver PPARδ levels detected by real-time PCR. The results show that 4-week-pretreatment with SIS3 (2.5 mg/kg.bw) inhibited p-Smad3 but increased PPARδ expression in liver tissues in *db/db* mice. Data represent the mean ± SEM for at least 6 mice per group. * *p* < 0.05, ** *p* < 0.01, **** *p* < 0.0001 versus normal db/m mice. ^#^ *p* < 0.05, ^##^ *p* < 0.01, ^###^ *p* < 0.001 compared with the control-treated *db/db* mice (vehicle). Scale bar, 50 μm. SIS3 treatment dose: S2.5 = 2.5 mg/kg.bw.

We investigated the therapeutic effects of SIS3 on hepatic PPARδ expression in *db/db* mice. The results show that SIS3 treatment effectively reduced hepatic Smad3 phosphorylation (p-Smad3) with the trend of a decrease in Smad3 mRNA expression, which was associated with an increase in hepatic PPARδ expression in *db/db* mice (Figure 6 and Figure S4) as determined by real-time PCR and western blotting analysis.

### 2.6. SIS3 Treatment Inhibits Fatty Liver and Reduces Lipid Accumulation by Blocking Smad3-Mediated Inhibition of PPARδ Expression In Vivo and In Vitro

**Figure 7:**
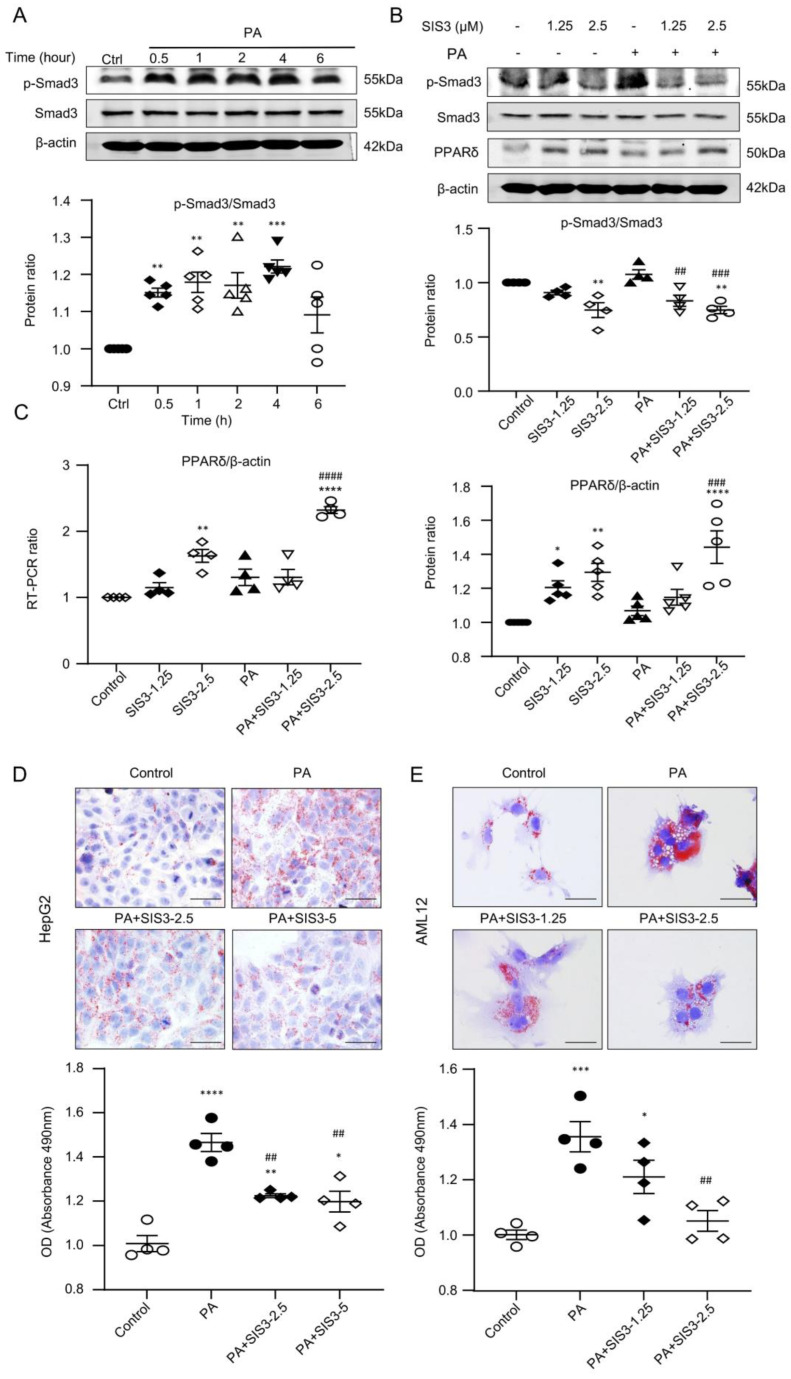
SIS3 treatment decreased lipid accumulation but increased PPARδ expression in hepatic AML12 cells in vitro. (**A**) Western blot analysis of Smad3 activation (p-Smad3) in response to PA stimulation (200 μM). (**B**) Inhibitory effect of SIS3 on PA-induced Smad3 activation (p-Smad3) and PPARδ expression by western blot analysis. (**C**) Inhibitory effect of SIS3 on PA-induced mRNA expression by RT-PCR. (**D**,**E**) Oil red O staining showed an inhibitory effect of SIS3 on PA-induced lipid accumulation in HepG2 and AML12 cells. The results show that treatment with SIS3 (2.5 μM) significantly inhibited p-Smad3 while increasing PPARδ mRNA and protein expression in AML12 cells. Each dot represents one independent experiment, and results are the mean ± SEM per group. * *p* < 0.05, ** *p* < 0.01, *** *p* < 0.001, **** *p* < 0.0001 versus the control. ^##^ *p* < 0.01, ^###^ *p* < 0.001, ^####^ *p* < 0.0001 versus the PA stimulation. The representative images are from at least three independent experiments. Scale bar, 100 μm.

In *db/db* mice, immunohistochemical staining, western blotting, and real-time PCR detected that the development of fatty liver in *db/db* mice was associated with marked activation of Smad3 and the loss of PPAR expression, which was blocked by SIS3 treatment (Figure 6). We next investigated the potential mechanism through which SIS3 treatment inhibits diabetic fatty liver in *db/db* mice and reduces lipid accumulation in hepatocytes under high-palmitic-acid (PA) conditions. In vitro, we found that the addition of PA to AML12 cells was capable of inducing the activation of Smad3 signaling by phosphorylation in as early as 30 min (Figure 7A), which was associated with the significant inhibition of PPARδ expression at both mRNA and protein levels (Figure 7B,C) and the increased massive lipid accumulation in HepG2 and AML12 cells (Figure 7D,E). Treatment with SIS3 at a dose of 2.5 μM significantly blocked PA-induced Smad3 phosphorylation while largely restoring PPARδ expression (Figure 7B,C), which was associated with the inhibition of lipid accumulation in HepG2 and AML12 hepatic cells (Figure 7D,E).

## 3. Discussion

By genetically deleting Smad3, we detected that Smad3 may be a critical mediator in the development of diabetic dyslipidemia and fatty liver in *db/db* mice. This was supported by the findings that *db/db* mice lacking Smad3 were protected against the development of diabetic dyslipidemia and fatty liver, showing normal serum levels of TG, TC, LDL-C, LDL-C/HDL-C, and TC/HDL-C without detectable lipid accumulation in the liver tissues in Smad3 KO-*db/db* mice. It has been shown that Smad3 plays an essential role in metabolic abnormalities in *db/db* mice as evidenced by the findings that *db/db* mice null for Smad3 are protected against hyperglycemia, obesity, glucose intolerance, and insulin resistance [13]. The results from the present study identify a new pathogenic role of Smad3 in diabetic dyslipidemia and NAFLD.

In the present study, we uncovered that Smad3 may mediate dyslipidemia and NAFLD by suppressing the expression of hepatic PPARδ. This was supported by the finding that Smad3 KO-*db/db* mice were protected against the development of diabetic dyslipidemia and fatty liver, which was associated with upregulation of PPARδ. It is well known that the PPAR family, including PPARα, PPARδ, and PPARγ, act as intracellular lipid sensors and regulate gene expression in response to lipid ligands [20,21]. Of them, PPARδ exerts a beneficial role in glucose and lipid metabolism, as well as NAFLD [23,24,25]. This is demonstrated by the findings that the overexpression of hepatic PPARδ can increase liver glucose utilization while changing the lipid profiles toward an increased ratio of monounsaturated to saturated fatty acids [25]. Many studies have also revealed that the activation of PPARδ attenuates hepatic steatosis by enhanced fat oxidation, reduced lipogenesis, and improved insulin sensitivity [26,27]. Thus, PPARδ activation has been shown to prevent obesity and to be an effective therapeutic strategy for the treatment of NAFLD, dyslipidemia, insulin resistance syndrome, and cardiovascular diseases [28,29,30,31,32]. It has been reported that Smad3 can bind C/EBP to inhibit the expression of PPARγ and PPARδ [33,34]. Thus, Smad3 plays an essential role in the inhibition of inflammation-induced PPARδ expression [35]. Smad3 KO mice exhibit an upregulation of PPARδ and its related genes, such as uncoupling protein (UCP) 2, UCP3, and acyl-CoA oxidase in adipose tissues, and are protected from diet-induced obesity and diabetes with improved glucose tolerance and insulin sensitivity [35,36]. In the current study, we discovered that TFs including *NFKB1, RELA, SPI1, JUN, ETS1* and *E2F1* were enriched in the liver tissues of Smad3KO-*db/db* mice through RNA-seq. The interaction of these pathways may be the factor regulating the change of PPARδ expression. Deletion of Smad3 from *db/db* mice may upregulate hepatic PPARδ and protect against the development of dyslipidemia and fatty liver in *db/db* mice. Our in vitro study also supported these notions, with the addition that PA was able to induce the activation of Smad3 signaling while inhibiting PPARδ expression, resulting in massive lipid accumulation in hepatocytes, which were blocked by a Smad3 inhibitor SIS3. Thus, restored PPARδ activities could be a mechanism through which Smad3 deficiency protects against the development of diabetic dyslipidemia and NAFLD in *db/db* mice.

Importantly, the present study also demonstrated that targeting Smad3 may represent a novel therapeutic potential for diabetic dyslipidemia and fatty liver, as the treatment of *db/db* mice with SIS3 resulted in a marked inhibition of Smad3 signaling and thus largely improved glucose tolerance, dyslipidemia, and NAFLD. This finding is consistent with a previous study that found that the systemic blockade of TGF-β signaling with antibody can protect against high-fat-diet-induced diabetes, obesity, and hepatic steatosis [36]. The protection against the Smad3-mediated inhibition of PPARδ expression could be a mechanism by which SIS3 treatment attenuates diabetic dyslipidemia and fatty liver.

It should be pointed out that antioxidants may also interfuse the blood glucose levels measured by the glucose meter [37]. It is unclear whether SIS3 treatment can suppress reactive oxygen species (ROS) production in diabetes and elevate circulating antioxidants. The previous study also showed that TGF-β1 increased the production of ROS by impairing mitochondrial function and inducing nicotinamide adenine dinucleotide phosphate (NADPH) oxidases [38,39]. Thus, the role of Smad3 in ROS production and the antioxidant effect of SIS3 on hyperglycemia need further investigation.

It should also be pointed out that the current study was based on the animal model. The lack of human data limits the translation of this finding to patients clinically. Although we have demonstrated the therapeutic effects of SIS3 on diabetic dyslipidemia and NAFLD in *db/db* mice, more studies in diabetic patients and human primary hepatocytes are needed.

## 4. Materials and Methods

### 4.1. Animals

All animal husbandry and animal experiments in this study were approved by the Animal Experimentation Ethics Committee of the Chinese University of Hong Kong (Ref No.: 17-559 in DH/SHS/8/2/1 Pt. 4) and confirmed to follow the regulations. All animal works were conducted in the Laboratory Animal Services Centre of The Chinese University of Hong Kong and Animal Unit of Prince of Wales Hospital. Four genotypes of mice (Smad3 WT-db/m, Smad3 KO-db/m, Smad3 WT-*db/db*, Smad3 KO-*db/db*) were generated by crossing Smad3 +/− mice to db/m mice and genotyped as described in our previous study [13,14,15]. All mice were bred and housed under specific pathogen-free (SPF) condition in a temperature-controlled room (25 °C) with a 12 h dark/light cycle setting. Groups of 6–8 mice from each of the four genotypes were used for experiments. All mice were sacrificed at week 10 by an intraperitoneal (i.p.) injection of phenobarbital. Blood and tissues were collected for various analyses as described below.

### 4.2. SIS3 Treatment

To determine an optimal dose of SIS3 for treatment of *db/db* mice without cytotoxicity, groups of 6–8 male db/m or *db/db* mice were i.p.-injected with SIS3 (Selleck Chemicals, Houston, TX, USA; cat# S7959) at dosages of 1.25, 2.5, and 5 mg/kg.bw/day, from the age of 4 weeks to 12 weeks, and then the optimal dose of 2.5 mg/kg.bw/day for treating *db/db* mice was determined based on histological observations and biochemical detection. Control-treated *db/db* mice (vehicle) were given the solvent (0.9% NaCl supplementary with 0.1% DMSO and 5% Tween 80). In addition, groups of 6–8 age-matched normal db/m and *db/db* mice were, respectively, used as normal and untreated-disease controls. All mice were sacrificed at week 12 by an i.p. injection of phenobarbital. Blood and tissues were collected following an overnight (12 h) fast. The effect of SIS3 on the development of T2D, dyslipidemia, and fatty liver was investigated at 12 weeks, including measures of liver weight-to-body weight ratio, fasting blood glucose (FBG) and serum lipid levels, the intraperitoneal glucose tolerance test (IPGTT), and the intraperitoneal insulin tolerance test (IPITT). The results were compared with the vehicle mice, the age-matched normal db/m and *db/db* mice without any treatment. All experimental protocols were approved by the Animal Experimentation Ethics Committee, the Chinese University of Hong Kong.

### 4.3. Fasting Blood Glucose, Body Weight, and Weight of Liver Tissues

Fasting blood glucose levels were measured by an Accu-Chek glucose meter (Roche Diagnostics, Indianapolis, IN, USA) in all mice after fasting overnight (12 h). For body weight measurements, mice were individually weighed at the indicated time points. In addition, liver tissues were dissected and weighed in individual mice sacrificed at 12 weeks of age.

### 4.4. Glucose and Insulin Tolerance Tests

For glucose tolerance tests (IPGTT), mice were fasted overnight (12 h) and given an i.p. injection of glucose (1 mg/g body weight). Tail tip blood glucose levels were determined at 0, 15, 30, 60, and 120 min after injection. For insulin tolerance tests (IPITT), mice were fasted for 5 h and given an i.p. injection of insulin (1 U/kg body weight). Blood glucose levels were determined at 0, 15, 30, 60, and 120 min after injection.

### 4.5. Hematoxylin and Eosin Staining and Immunohistochemistry Staining

Hematoxylin and Eosin (H&E) staining and immunohistochemical staining were performed on methyl-canoy-fixed, paraffin-embedded tissue sections (3 μm) as described in previous studies [13,14,15]. Briefly, for immunohistochemical staining, tissue sections were incubated with primary antibodies against phosphor-Smad3 (p-Smad3) (Abcam, Cambridge, MA, USA, cat# ab52903), PPARδ (Abcam, cat# ab23673), at 4 °C overnight, followed by incubation with appropriate HRP-labeled secondary antibodies. After being developed with 3,3′-diaminobenzidine (DAB) to produce a brown color, sections were counterstained with hematoxylin. The stained sections were then quantified by the quantitative image software (Image-Pro Plus 7.0, Media Cybernetics, Rockville, MD, USA) as previously described [13,14,15].

### 4.6. Oil Red O Staining

Liver frozen sections (6–7 μm) were fixed in 4% paraformaldehyde for 30 min and rinsed with 60% isopropanol for 15 s and then stained with freshly prepared Oil Red O working solution (Sigma-Aldrich Corp, St Louis, MO, USA; cat# O1391) for 15 min. After being rinsed with 60% isopropanol for 1–2 s, sections were counterstained with hematoxylin. The staining-positive area was quantitated with Image J software (version 1.48, NIH, Bethesda, MD, USA).

### 4.7. BODIPY (493/503) Staining

Liver frozen sections (5 μm) were fixed in 4% paraformaldehyde for 30 min and rinsed with PBS and stained with BODIPY (493/503) (Thermo Fisher Scientific, Cleveland, OH, USA, cat# D3922) as described in a previous study [40]. The stained sections were then viewed under fluorescence microscopy and quantitatively analyzed using the Image-Pro plus 7.0 software and GraphPad Prism 7 software (GraphPad Software, San Diego, CA, USA).

### 4.8. Filipin III Staining

For cholesterol staining, liver frozen sections (5 μm) were fixed in 4% paraformaldehyde for 30 min, rinsed with PBS, and incubated with 1.5 mg/mL glycine in PBS for 10 min at room temperature to quench the paraformaldehyde. The slices were then stained with 50 μg/mL Filipin III (Sigma, cat# F4767) working solution for 2 h at room temperature, viewed under fluorescence microscopy, and quantitatively analyzed using the Image-Pro plus 7.0 software and GraphPad Prism 7 software.

### 4.9. Measurement of Blood Lipids and Liver Functions

Lipid parameters, including serum TC, serum TG, serum HDL-C, serum LDL-C, and liver TC and TG, as well as liver functions such as serum aspartate aminotransferase (AST), alanine aminotransferase (ALT), and lactate dehydrogenase (LDH), were determined by following the manufacturer’s instructions (Nanjing Jiancheng Bioengineering Institute, Nanjing, China). In addition, liver cholesteryl ester (CE), free cholesterol (FC), and TC were examined using the assay kits according to the manufacturer’s instructions (Abcam, cat# ab65359).

### 4.10. Western Blotting Analysis

Total protein from liver tissues or cultured hepatocytes were extracted by RIPA, and western blotting analysis was performed as previously described. The primary antibodies used in this study included: p-Smad3 (Abcam, cat #ab52903), Smad3 (Abcam, cat #ab28379), β-actin (Santa Cruz Biotechnology, cat #sc-69879), and PPARδ (Thermo Fisher Scientific, cat #PA1-823A). After being incubated with the primary antibody at 4 °C overnight, the membrane was stained with the LI-COR IRDye 800-labeled secondary antibodies (1:3000, Rockland Immunochemicals, Gilbertsville, PA, USA) for 1 h. The signals were detected with the Odyssey Infrared Imaging System (Li-COR Biosciences, Lincoln, NA, USA) and quantitated with Image J software (version 1.48, NIH). The intensity of the protein band was normalized against β-actin or total proteins as stated in the studies and expressed as the mean ± SEM.

### 4.11. RNA Extraction and Quantitative Real-Time PCR

RNA was extracted from mouse liver or cultured hepatocytes using Trizol (Invitrogen, Carlsbad, CA, USA; cat# 15596026), according to the manufacturer’s instructions, and real-time PCR was performed using Bio-Rad IQ SYBR Green Supermix with Option 2 (Bio-Rad, Hercules, CA, USA) as described previously [13,14,15,16]. The primers used in this study included: PPARα forward 5′-CTGGTCTTAACCGGCCCAAT-3′ and reverse 5′-GTGCACATAGCCAGAAGGGT-3′, PPARγ forward 5′-TTGCTGTGGGGATGTCTCAC-3′ and reverse → 5′-AACAGCTTCTCCTTCTCGGC-3′, PPARδ → forward 5′-GCTGCTGCAGAAGATGGCA-3′ and reverse 5′-CACTGCATCATCTGGGCATG-3′, β-actin forward 5′-AGAGGGAAATCGTGCGTGAC-3′ and reverse 5′-CAATAGTGATGACCTGGCCGT-3′, Smad3 forward 5′-GTCTCAGAGTGTTCACAGGAAGCA-3′ and reverse 5′-TATACATCAGGGTTGTGGTGCCAG-3′. The relative level of the detected gene was normalized with the internal control β-actin by the delta-delta Ct method and expressed as mean ± SEM.

### 4.12. Cell Culture

HepG2 human cancer cells (ATCC HB-8065) were cultured in DMEM (Gibco, Life Technologies, Carlsbad, CA, USA; cat# 11965092) and supplemented with 10% fetal bovine serum (FBS) (Gibco, Life Technologies; cat# 16140071) and 1% penicillin and streptomycin (Gibco, Life Technologies; cat# 15140122) in 5% CO2 at 37 °C. The HepG2 cells were pretreated with SIS3 at dosages of 0, 2.5, and 5 μM (Selleck Chemicals, Houston, TX, USA; cat# S7959) or DMSO for 1 h, respectively, and then incubated with 200 μM Palmitic acid (PA) (Sigma, USA; cat# P0500) or BSA-Fatty Acid Free (Sigma, St Louis, MO, USA; cat# A7030-50 G) for an additional 24 h. The cells were stained for Oil Red O. In addition, the same experimental protocol was also carried out in AML12 cells (ATCC CRL-2254), an immortalized normal mouse hepatocyte (a kind gift from Professor Jun Yu from the Chinese University of Hong Kong). The cells were stained for Oil Red O (cultured 24 h) or subjected to real-time PCR (cultured 12 h) and western blotting analysis (cultured 24 h). To determine PA-induced Smad3 phosphorylation, AML12 cells were cultured with 200 μM PA for 30 min, 1, 2, 4, and 6 h, respectively, for western blotting analysis.

### 4.13. RNA Sequencing and Functional Enrichment Analysis

RNA sequencing and the downstream data analysis were described previously [13,16]. RNA samples from 8 mice per group (Smad3 WT-db/m, Smad3 KO-db/m, Smad3 WT-*db/db*, Smad3 KO-*db/db* mice) were pooled into one sample for RNA-seq. Briefly, one sample-pooled sequencing library was prepared, and RNA-seq was conducted as previously described [41]. First, total RNA was isolated from liver tissues with Trizol according to the manufacturer’s instructions. Then genomic DNA was removed by adding Dnase I digestion, and the quality and purity of RNA were checked using the NanoDrop 1000 Spectrophotometer. The libraries were 250–300 bp insert and strand-specific (Ribo-ZeroTM Magnetic Kit) at Novogene Company Limited (Hong Kong, China). The Agilent 2100 Bioanalyzer System was used to check the sample integrity. The RNA-seq was performed by an Illumina Genome Analyzer (HiSeq 2000 Sequencing System) (Illumina, San Diego, CA, USA). FastQC (v0.11.5) was used to ensure the reliability of the 51~95 million 101 bp paired-end raw sequencing reads per sample. Reads were mapped to the mm10 assembly using HISAT2 (v2.0.5), and feature counting was conducted by the tool htseq-count. Count-based differential expression analysis of RNA-seq was performed using DESeq2 (v1.16.1). For DESeq2, single-function DESeq that encapsulates the standard steps for conducting differential expression analysis was used as previously described [42]. Differential expression genes (DEGs), significantly enriched Kyoto Encyclopedia of Genes and Genomes (KEGG) pathways, and gene ontology (GO) analysis data performed using DAVID were defined as FDR < 0.1.

### 4.14. Statistical Analysis

All the data are expressed as mean ± SEM. All statistical analysis between two groups were performed by a one-way analysis of variance (ANOVA), followed by a Newman–Keuls multiple comparisons test using Prism 7.0 (GraphPad Software, San Diego, CA, USA). Moreover, *p*-values below 0.05 were considered significant.

## Figures and Tables

**Table 1:** AUC of FBG, IPGTT and IPITT of the four groups of mice at 12 weeks.

Group (*n* = 6)	AUC-FBG (mmol/L × min)	AUC-IPGTT (mmol/L × min)	AUC-IPITT (mmol/L × min)
db/m	61.01 ± 8.39	45.14 ± 6.49	11.44 ± 1.73
db/db	128.33 ± 19.37 *p* < 0.0001 ****	164.70 ± 7.17 *p* < 0.0001 ****	102.25 ± 14.48 *p* < 0.0001 ****
db/db + vehicle	128.82 ± 13.09 *p* < 0.0001 ****	165.20 ± 8.14 *p* < 0.0001 ****	109.62 ± 10.77 *p* < 0.0001 ****
db/db + S2.5	83.08 ± 2.58 *p* < 0.05 * p < 0.0001 ^####^	118.47 ± 6.25 *p* < 0.0001 **** p < 0.0001 ^####^	80.01 ± 11.57 *p* < 0.0001 **** p < 0.0001 ^####^

* *p* < 0.05, **** *p* < 0.0001 versus normal db/m mice. ^####^ *p* < 0.0001 compared with the vehicle. SIS3 treatment dose: S2.5 = 2.5 mg/kg.bw.

**Table 2:** LDH levels and body weights of mice in the four groups at 12 weeks.

Group (*n* = 8)	LDH (U/L)	Body Weight (g)
db/m	774.27 ± 88.11	25.13 ± 1.64
db/db	801.11 ± 61.97 *p* < 0.0001 ****	48.75 ± 4.17 *p* < 0.0001 ****
db/db + vehicle	780.17 ± 68.26 *p* < 0.0001 ****	45.88 ± 3.27 *p* < 0.0001 ****
db/db + S2.5	757.06 ± 103.62 *p* < 0.0001 ****	46.75 ± 1.83 *p* < 0.0001 ****

**** *p* < 0.0001 versus normal db/m mice. SIS3 treatment dose: S2.5 = 2.5 mg/kg.bw.

**Table 3:** ALT and AST levels of mice in the four groups at 12 weeks.

Group (*n* = 6)	ALT (IU/L)	AST (IU/L)
db/m	10.82 ± 2.29	17.36 ± 4.65
db/db	93.17 ± 24.04 *p* < 0.0001 ****	170.18 ± 48.17 *p* < 0.0001 ****
db/db + vehicle	88.06 ± 21.29 *p* < 0.0001 ****	169.81 ± 39.77 *p* < 0.0001 ****
db/db + S2.5	41.19 ± 11.49 *p* < 0.01 ** p < 0.001 ^###^	59.17 ± 15.76 *p* < 0.05 * p < 0.0001 ^####^

* *p* < 0.05, ** *p* < 0.01, **** *p* < 0.0001 versus normal db/m mice. ^###^ *p* < 0.001, ^####^ *p* < 0.0001 compared with the vehicle. SIS3 treatment dose: S2.5 = 2.5 mg/kg.bw.

## Data Availability

All data that support the findings of this study are reported in the main paper and Supplementary Materials. RNA-seq raw data was deposited to Sequence Read Archive (SRA), NCBI. BioProject ID: PRJNA932600 (http://www.ncbi.nlm.nih.gov/bioproject/932600, accessed on 8 February 2023). The data will be released on 31 August 2023 (or upon publication, whichever is first).

## Supplementary Materials

The following supporting information can be downloaded at: https://www.mdpi.com/article/10.3390/ijms241411396/s1.

## Abbreviations

ALT: alanine aminotransferase; AST: aspartate aminotransferase; BP: biological process; CE: cholesterol ester; CVD: cardiovascular diseases; DEG: differential expression gene, DM: diabetes mellitus; DAB: 3,3′-diaminobenzidine; FBG: fasting blood glucose; FBS: fetal bovine serum; FC: free cholesterol; GO: gene ontology; HDL-C: high-density lipoprotein cholesterol; I.P.: intraperitoneal injection; IPGTT: intraperitoneal glucose tolerance test; IOD: integrated option density; IPITT: intraperitoneal insulin tolerance test; Kg. bw: kg body weight; KEGG: Kyoto Encyclopedia of Genes and Genomes; KO: knockout; LDH: lactate dehydrogenase; LDL-C: low-density lipoprotein cholesterol; NADPH: nicotinamide adenine dinucleotide phosphate; NAFLD: non-alcoholic fatty liver disease; OD: optical density; PA: palmitic acid; p-Smad3: phospho-Smad3; PPAR: peroxisome proliferator-activated receptor; RNA-seq: RNA sequencing; ROS: reactive oxygen species; SEM: the standard error of the mean; SPF: specific pathogen-free; STZ: streptozotocin; S3: Smad3; T2DN: Type-2 diabetic nephropathy; TC: total cholesterol; TFs: transcription factors; TG: triglycerides; T2D: type 2 diabetes. TGF-β: transforming growth factor-β; UCP: uncoupling protein; WT: wild-type.
