# Supplementary material for: Smad3 Mediates Diabetic Dyslipidemia and Fatty Liver in db/db Mice by Targeting PPARδ
Source: Int J Mol Sci. 2023 Jul 13;24(14):11396. doi: 10.3390/ijms241411396 (PMC10380492; doi:10.3390/ijms241411396)
Supplement: Supplementary file 1 [file ijms-24-11396-s001.zip › ijms-2473499-supplementary.pdf]

Supplementary Figures

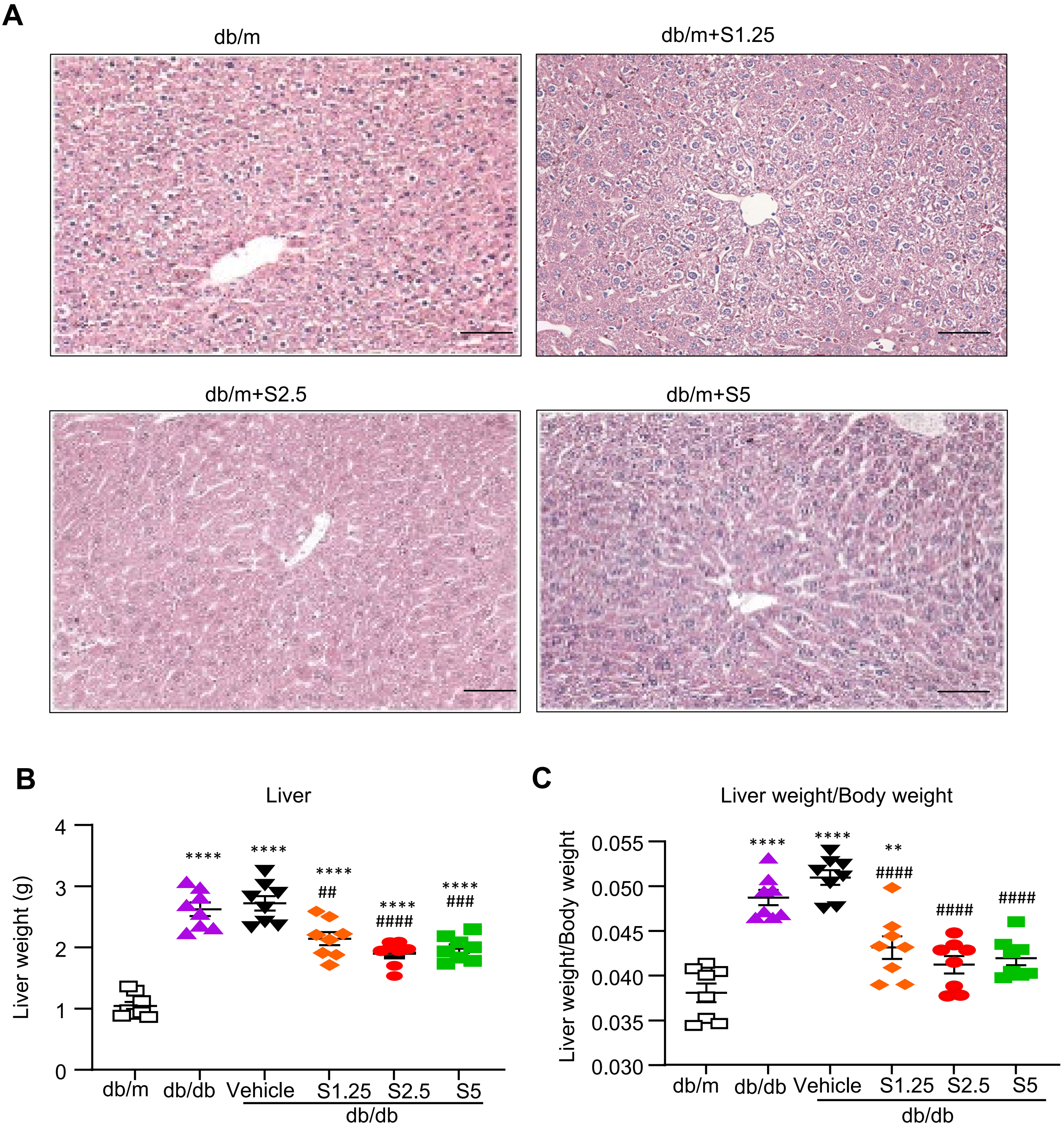

Figure S1. SIS3 treatment inhibits liver weight with an optimal dosage at 2.5 mg/kg without systemic toxicity in db/m and db/db mice. (**A**) H&E staining of liver tissues from db/m mice. (**B**) Liver weight. (**C**) Liver weight against body weight. Data represents the mean ± SEM for at least 6 mice per group. $^{**}P$ <0.01, $^{****}P$ <0.0001 versus normal db/m mice. $^{\#\#}P$ <0.01, $^{\#\#\#}P$ <0.001, $^{\#\#\#\#}P$ <0.0001 compared with vehicle-treated db/db mice. Scale bar, 50 μm. Dose dependent treatment of SIS3: S1.25=1.25

mg/kg.bw, S2.5=2.5 mg/kg.bw, S5=5 mg/kg.bw.

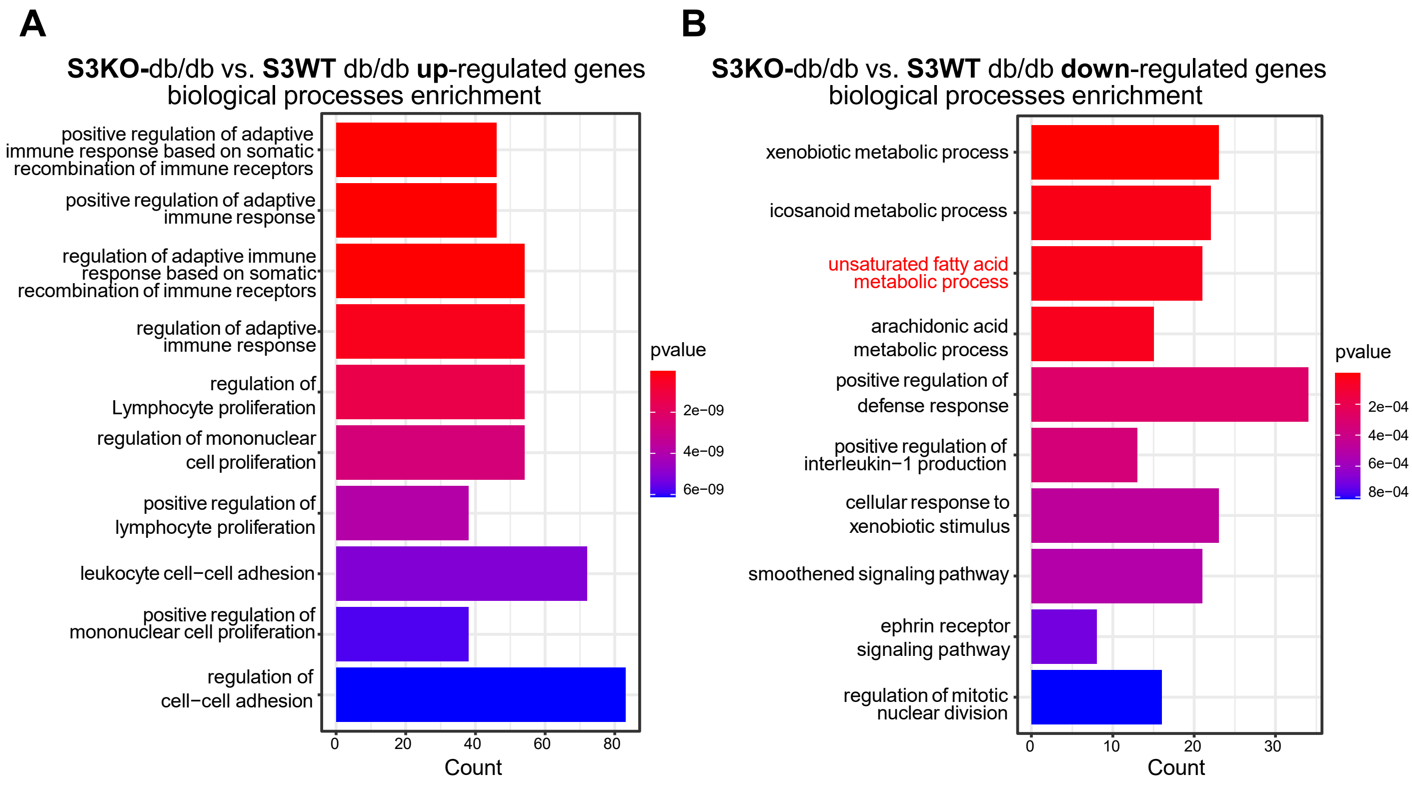

Figure S2. **Bioinformatic analysis of DEGs in livers by RNA-seq.** (**A-B**) The top 10 gene ontology (GO) biological process (BP) terms enrichment of upregulated and downregulated genes in livers of Smad3 KO-db/db relative to Smad3 WT-db/db mice showing that Smad3 KO-db/db downregulated genes are significantly enriched in unsaturated fatty acid metabolic process.

A

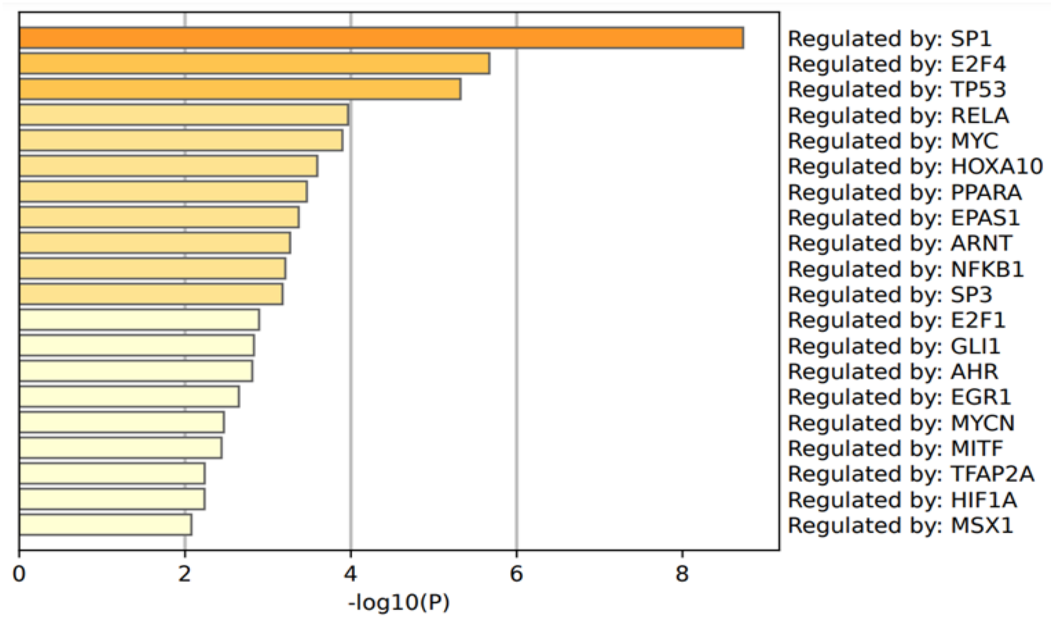

B

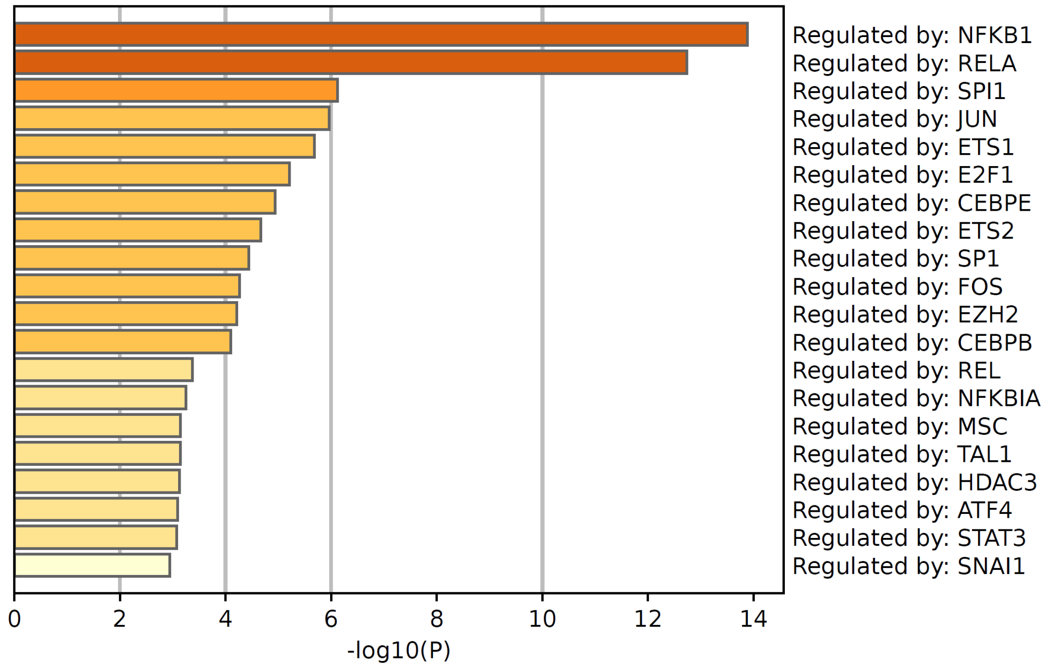

Figure S3. **TF-target enrichment was preformed based on the upregulated DEGs of Smad3-WT db/db mice or Smad3 KO db/db mice by Metascape.** (**A**) TF enrichment of upregulated DEGs of Smad3-WT db/db mice vs. Smad3-WT db/m. (**B**) TF enrichment of upregulated DEGs of Smad3-KO db/db vs Smad3-WT db/db mice.

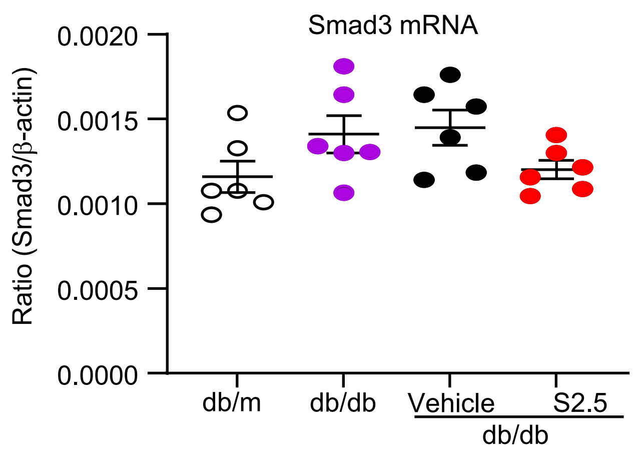

Figure S4. **Bioinformatic analysis of DEGs in livers by RNA-seq.** Liver Smad3 levels detected by real-time PCR. Data represents the mean ± SEM for at least 6 mice per group. SIS3 treatment dose: S2.5=2.5 mg/kg.bw.
